# Examining Teacher Concerns and Anxiety on the Implementation of a Universal Body Image Program

**DOI:** 10.3390/ijerph21040419

**Published:** 2024-03-29

**Authors:** Sarah G. Hidalgo, Taryn Henning, Francesca Gomez, Marisol Perez

**Affiliations:** 1 REACH Institute, Department of Psychology, Arizona State University, Tempe, AZ 85287-1104, USA; sgdahl@asu.edu; 2 Department of Psychology, Virginia Commonwealth University, Richmond, VA 23284-2018, USA; henningt@vcu.edu; 3 Department of Psychology, Arizona State University, Tempe, AZ 85287-1104, USA; fgomez16@asu.edu

**Keywords:** body image, prevention, implementation, youth, teacher, self-efficacy, body esteem, mental health, behavioral interventions, eating pathology

## Abstract

In the past 30 years, there have been numerous positive body image and eating disorder prevention programs targeting youth developed for school-based settings. Frequently, teachers are used as interventionists to increase dissemination, decrease costs relative to researchers, and increase scalability. However, little is known about teacher concerns and barriers that may hinder successful uptake and implementation. The current study recruited a total of 269 teachers who consented to implement a universal body image and appearance-related bullying and teasing prevention program in their classrooms as part of a randomized controlled trial. Teachers expressed some worry that they may say the wrong thing, and concern about feeling uncomfortable teaching the program due to their own body dissatisfaction. Teacher’s ethnicity, gender, years teaching, dieting behaviors and other weight control behaviors, and self-efficacy were not associated with concerns related to teaching the curriculum. Teachers with lower body esteem reported higher concerns and anxiety related to teaching a body image curriculum. In free response items, teachers worried about handling student comments that were beyond the scope of the curriculum. Teacher self-efficacy was the only variable associated with the number of program sessions implemented. Findings suggest avenues to increase implementation.

## 1. Introduction

The dissemination and implementation of evidence-based interventions recommends assessing provider concerns and barriers to implementation [1]. Universal mental health prevention programs developed for school settings have the ability to reach large numbers of youth in a cost-effective and scalable manner [2]. Within the last several decades, there has been an increase in the development of preventive evidence-based programs aimed at reducing eating disorder symptoms and body image concerns among children and adolescents [3]. Many of these body image interventions occur in classroom-based settings and have been shown to produce reductions in body dissatisfaction [4], with some demonstrating sustained improvement in body image concerns through follow-up [5]. Importantly, adopted and widely distributed evidence-based prevention programs tend to vary in quality of program implementation and can, at times, fail when implemented in real-world settings [6]. Although there is research addressing the effectiveness of body image prevention programs, there is a lack of attention to potential barriers precluding implementation of these interventions in school settings. Understanding and identifying potential barriers to the implementation of prevention programs, including teacher-led body image prevention programs, is necessary to increasing the use and effectiveness of these programs.

The majority of teacher-implemented school-based programs addressing body image concerns focus on adolescents, with relatively fewer available for pre-adolescent children. In a recent systematic review conducted by Kusina and Exline, researchers examined 34 classroom-based intervention programs designed to reduce body dissatisfaction and promote positive body image among youth aged 12–18 [7]. In their review, researchers identified notable variations among these programs, including differences in intervention methods, interventionists, and their impact on outcomes. Kusina and Exline reported that universal programs, as compared to alternate approaches, result in increased body image [7] in adolescents consistent with previous research [4]. In addition, program outcomes did not differ across studies that used researchers versus teachers as the interventionist. However, there is one study among adolescents that found significant improvements in body image when teachers led the program as opposed to research staff [2]. In contrast to adolescents, there is a paucity of research on school-based body image programs for pre-adolescent children. Existing research finds that school-based body image programs yield body image improvements post-intervention [8,9,10,11], at times yielding more benefits for girls than boys [8] and with differential maintenance of outcomes at follow-up [8,12]. Similar to findings for adolescents, teacher-led programs are effective in increasing body image and self-esteem among 5-to-8-year-old children [13] and decreasing internalization of thin ideals and eating disorder behaviors across 11-to-12-year-old children, with small to medium effect sizes [12]. Collectively, research across both childhood and adolescence suggests teachers can be effective at implementing body image programs in schools. This is important for dissemination of prevention programs as the use of teachers relative to researchers and mental health professionals is more cost-effective and makes it possible to disseminate at larger scale [2,14].

To date, there has been no research conducted on potential barriers to the implementation of teacher-led body image programs. However, school-based prevention programs for other psychological issues, such as bullying and teasing, physical activity, and violence, have explored the influence of teacher characteristics on program implementation. For example, in the Olweus Bullying Prevention Program, teachers are key in implementation of the program [15]. Empirical work has explored teacher characteristics such as demographics and self-efficacy, teacher perceptions of bullying as an issue in schools, teacher experiences with bullying, and school climate, as influencing the number of activities from the Olweus program implemented in the classroom [15]. Higher teacher self-efficacy, more frequent teacher experiences with bullying, and a greater perceived need for the program predicted higher numbers of programmatic activities implemented and greater teacher involvement in the curriculum overall [15]. In a review of school-based physical activity programs, higher teacher self-efficacy predicted implementation of a physical activity program in the classroom [16]. Further, the personal physical activity habits of teachers predicted teacher self-efficacy, which, in turn, predicted if the program was implemented in the classroom [16]. The implementation evaluation of a mental and social well-being promotion program found teacher characteristics such as teacher motivation was associated with program implementation and the overcoming of barriers and challenges that occur with program implementation [17]. In a teacher-led school-based violence prevention program, teacher self-efficacy predicted initial program implementation, and increases in self-efficacy across time predicted increases in program implementation across time as well [18]. Finally, in a review of 500 studies that implemented promotion and prevention programs for youths across physical health, mental health, academic, and development categories, the interventionist or provider characteristics that were consistently related to implementation were the recognition of a need for the program, the belief that the program would be beneficial, self-efficacy, and having the required skills to implement the program [1]. Collectively, these studies highlight the impact of interventionist characteristics in implementation and, specifically, self-efficacy consistently emerged as associated with program implementation across diverse school-based prevention programs.

Although there is no research on implementation, needs assessment surveys have been conducted that elucidate some teacher concerns related to body image. For example, early childhood and elementary school teachers reported wanting body image resources for promoting positive body image in children, how to deal with external influences such as messages from the media, and early detection and intervention strategies [13,19]. Although limited, this empirical work suggests teachers perceive a need, but have a lack of training for handling the issues that arise and promoting a positive body image in the classroom. This potentially suggests that teacher self-efficacy and teacher concerns may play a role in the implementation of a body image program.

The current study aimed to address the gap in the literature related to teacher characteristics and perceptions related to implementing a school-based body image program. The current study explored the relationships between teacher characteristics such as gender, years teaching, self-efficacy, teacher concerns with implementing a universal school-based body image program, and the number of weeks the program lessons were implemented. Consistent with previous research, we hypothesized that higher levels of self-efficacy and more years in teaching would be associated with fewer teacher concerns and more program lessons implemented (i.e., more program content covered in class). Exploratory analyses were conducted to examine if teacher’s own body esteem, dieting behaviors, or other weight control behaviors were associated with teacher concerns or number of program lessons implemented. Previous work has found that both parents and educators are concerned with their own body image concerns influencing the children’s body image [13,19]. Thus, we hypothesized that higher teacher body esteem scores would be related with fewer teacher concerns and more program implementation; conversely, we hypothesized that greater dieting or other weight control behaviors would be associated with a greater number of teaching concerns and less program implementation.

## 2. Materials and Methods

### 2.1. Participants and Procedures

Teachers were recruited online from October 2020 to June of 2022 via professional and social media platforms to participate in a randomized controlled trail of Amazing Me, a universal prevention program aimed at increasing positive body image and decreasing appearance-based bullying and teasing in 4th and 5th grade students. A total of 796 teachers expressed interest in participating in the study. Eligibility criteria included teaching 4th and 5th grade students within the United States. The teachers were randomized to an intervention or control group and sent an online survey via Qualtrics as the baseline. All of the variables in the current study (except for program implementation) were assessed during the baseline survey. In total, 269 teachers completed the baseline survey; however, six teachers completed the survey twice with the duplicate survey removed resulting in 124 intervention teachers and 139 control condition teachers, resulting in a total sample of 263 teachers. As displayed in Table 1, the teachers were between the ages of 22 and 70 years (M = 41.91, SD = 9.65), with 89.6% of them reporting as female, 3.5% male, 0.4% gender non-conforming, and 6.6% preferring not to answer. Teachers could select multiple race and ethnic identities and reported identifying as 3.1% Asian American, 11.6% Black or African American, 6.2% Latine, 69.1% White, 4.2% Multiracial, and 5.0% as ‘other’.

After completing online teacher surveys, teachers administered online student surveys within their classrooms. Then, teachers in the intervention condition administered the program. Six weeks later, teachers in both conditions and their students completed online surveys again (i.e., post-intervention survey), and at follow-up. Administration of the online surveys took approximately 15 to 20 min. The teachers were compensated with $100 in classroom supplies per survey completed. Teachers in the control condition were given the curriculum after follow-up. This study was approved by the university’s institutional review board. All teachers provided written consent, all parents were notified of the study and given the opportunity to opt their child out of the study, and all children provided written assent. All youth materials were written at a 3rd and 4th grade reading level.

A community-based participatory approach was used in the design of the curriculum. For brevity, we refer to this intervention as a body image program or curriculum. A design team consisting of two 4th and 5th grade teachers, three children between 9 and 11 years old, and three experts on prevention programs for positive body image, developed the curriculum. Teachers within the intervention condition were provided with ten lessons (five activities that focused on promoting positive body image and five activities that focused on reducing appearance- and weight-based bullying and teasing), each approximately 45 min in duration. It was recommended that teachers implement six lessons, approximately once a week. Nine family activities were provided so that teachers could send materials home to extend the learning. Seventeen percent of teachers submitted audio-recordings of their classroom lessons (note: the targeted goal was to receive recordings from 20% of teachers). Undergraduate research assistants underwent 12 h of training for coding fidelity and teacher competence. Fidelity assessment and teacher competence followed Stice, Rohde, Gau, and Shaw’s [20] fidelity assessment procedure and scoring, which have also been used by previous school-based body image programs [2,5]. Each lesson was rated on how closely the teacher followed the lesson plan using a 10-point Likert scale from 1 = No adherence, section was skipped to 10 = perfect. All material was presented as written. Teacher competence included ratings on the teacher’s ability to explain the lesson clearly, appropriate pace, overall tone, organization, student engagement, etc., on a scale from 1 = poor to 10 = superior. Inter-rater reliability across research assistants was good (ICCs = 0.69–0.95). Teacher fidelity to the lessons as designed was good (M = 6.31, SD = 2.04), with 60.6% of the intervention teachers covering 70% or more of each lesson. Teachers were rated as good to excellent/superior in competence (M = 6.59, SD = 1.40). 

### 2.2. Measures

#### 2.2.1. Demographics

Teachers self-reported at baseline their gender identity from the following options: cisgender man, cisgender woman, transgender man, transgender woman, gender non-conforming, two spirited, not sure, prefer not to answer, and not listed above. We acknowledge that the terms and language around gender minoritized people is constantly evolving, and how we have classified gender identity is not fully inclusive of the complexities of gender identities. Teachers self-reported race and ethnicity at baseline and were able to check all identities that applied including Black, American Indian, Asian, Latino, Middle Eastern, Native Hawaiian, White, I prefer not to answer, and other.

#### 2.2.2. Years Teaching

Teachers reported the number of years they had spent teaching via a text box response at baseline. The data were transformed to numeric responses that ranged from less than one year to 37 years. 

#### 2.2.3. Body Esteem

The Body Esteem Scale for Adults and Adolescents (BESAA) [21] assesses teachers’ general feelings about appearance, weight satisfaction, and perception of how others view one’s appearance and body, and was administered as part of the baseline survey. The 23 items are rated on a 5-point Likert scale from 1 (Never) to 5 (Always). Sample items include “My looks upset me” and “I like what I look like in pictures”. Total scores are averaged where higher scores indicate greater body esteem (i.e., liking or being satisfied with one’s own body). The internal consistency of item scores on the BESAA was α = 0.94 for the current sample.

#### 2.2.4. Dieting Behaviors

The restrained eating subscale of the Dutch Eating Behavior Questionnaire (DEBQ) [22] was administered to teachers at baseline. Sample items included, “Do you watch exactly what you eat?” and “Do you take into account your weight with what you eat?”. Participants reported how frequently they engaged in restrictive behaviors by responding on a 5-point scale ranging from 1 (Never) to 5 (Very Often). Item scores were averaged with higher scores indicating greater engagement in dieting behaviors. For the current sample, the internal consistency of the item scores was α = 0.89.

#### 2.2.5. Weight Control Behaviors

As part of the baseline survey, teachers were asked if they engaged in any of the following behaviors as a means to control their weight, shape, or intake of calories: exercising in a compulsive way, counting calories, avoiding certain foods, limiting the amount of calories consumed, skipping meals, taking diet pills, diuretic use, vomiting, laxative use, fasting, and meal replacements. A frequency count of behaviors endorsed was computed with higher scores indicating greater endorsement of weight control behaviors. 

#### 2.2.6. General Self-Efficacy

The New General Self-Efficacy Scale (NGSES) [23] consists of 8 items assessing how much people believe they can achieve their goals, despite difficulties assessed at baseline. The scale utilizes a 5-point rating scale raining from 1 (Strongly Disagree) to 6 (Strongly Agree). Items were adapted to apply to teaching tasks. For example, the item “I am confident that I can perform effectively on many different tasks” was adapted to “I am confident that I can perform effectively on many different teaching tasks”. Another example includes adapting an item from “I will be able to achieve most of the goals that I have set for myself” to “I will be able to achieve most of the teaching-related goals that I have set for myself”. The items were averaged, with higher scores indicating greater perception of teaching self-efficacy. For the current study, the internal consistency of the item scores was α = 0.92.

#### 2.2.7. Teaching Concerns

As part of the baseline survey, teachers were asked 7 items to assess concerns related to teaching a body image program. Using a 4-point rating scale, 1 (Not at all concerned) to 4 (Very concerned), teachers were asked to indicate how much concern they felt about the following: “I feel that I do not know enough about body image to teach this type of curriculum to others”; “I worry I might say the wrong thing”; “I have never done anything like this before and I don’t know what it will be like”; “I worry that others will find out that I am dissatisfied about my body”; “I sometimes feel dissatisfied about parts of my body and this makes me feel uncomfortable”; “Talking about body image makes me feel uncomfortable”; and “Discussing body image with 4th and 5th graders seem difficult”. The item scores were averaged with higher scores indicating greater teaching concerns. The internal consistency of the item scores was α = 0.76.

#### 2.2.8. Program Implementation among Intervention Teachers 

In the post-intervention survey, teachers reported on how many weeks they implemented the program, with options ranging from one to seven or more weeks. 

### 2.3. Data Analysis

All analyses were completed using SPSS version 28. Three teachers did not provide any data on the variables of interest and were removed from the analyses, resulting in a sample of 259 teachers. Across all of the variables in the study, less than five percent of the data were missing. The mean, standard deviation, skewness, kurtosis, and regression diagnostics were examined to ensure data were appropriate for regression.

#### 2.3.1. Analyses with the Full Sample of Teachers

To examine if teacher demographics, number of years teaching, teacher body esteem, dieting behaviors, weight control behaviors, and self-efficacy were associated with teacher concerns, a hierarchical regression equation was computed. In the first model, teacher gender and race/ethnicity were dummy coded and regressed on teacher concerns. In the second model, body esteem, dieting behaviors, weight control behaviors, self-efficacy, and number of years teaching were regressed on teacher concerns. 

At baseline, teachers were given the opportunity to provide written feedback on concerns they had implementing a body image program. The responses were grouped into themes and described.

#### 2.3.2. Analyses with Intervention Teachers

Among the teachers that were part of the intervention condition (*n* = 124), the number of weeks implementing the program was assessed. A hierarchical regression equation was computed with weeks implementing the program as the dependent variable. In the first model, teacher gender and race/ethnicity were entered. In the second model, body esteem, dieting behaviors, weight control behaviors, self-efficacy, number of years teaching, and teaching concerns were entered.

## 3. Results

### 3.1. Descriptive Analyses

As shown in Table 2, higher dieting and weight control behaviors were significantly related to higher teaching concerns. In contrast, greater teacher self-efficacy and body esteem were associated with fewer teaching concerns. There was no significant correlation between teaching concerns and the number of years teaching, gender, ethnicity, or the number of weeks the program was implemented. Program implementation was only positively correlated with teacher self-efficacy.

### 3.2. Teacher Concerns Related to Teaching a Body Image Program across a Full Sample of Teachers

An examination of the mean level of the teacher concerns scores revealed that the mean level of concerns is at 1.52 (SD = 0.44), indicating responses fell between not at all concerned and a little concerned. Table 3 displays the percentage of teachers that endorsed concerns for each item of the scale. As can be seen, 64.9% of teachers reported some concern with saying the wrong thing while teaching the program, including 13.5% who expressed moderate to very concerned. Similarly, 56% of teachers reported some concern related to being uncomfortable teaching the program due to their own body dissatisfaction, with 15.8% reporting moderate to significant concern. A little more than half (54.5%) of the teachers reported some concern due to inexperience teaching a program like Amazing Me, including 13.6% who expressed moderate concern to very concerned. 

Teachers were given the opportunity to provide written feedback on additional concerns with teaching a body image program, and 20 teachers provided written feedback. Ten teachers (50% of those who provided written feedback) expressed concerns over how their students would react to the program as well as the potential conversations and disclosure this program might facilitate. For example, one teacher expressed concern about “not knowing where the conversations will lead beyond the scope of the curriculum.” In addition, six teachers (30%) expressed concerns about parents’ reactions to the program and whether these would be negative. Two teachers (10%) expressed concerns about their own body image negatively affecting their ability to implement the program. For instance, one teacher stated, “Will my own body image taint my teaching on this topic?” Finally, two teachers (10%) expressed concerns about how their relationship with the students may impact the potency of the program.

### 3.3. Predictors of Teacher Concerns across Full Sample of Teachers 

As displayed in Table 4, Model 1 accounted for 3% of the total variance of teaching concerns (F = 3.72, *p* = 0.03). Teacher gender was significant when regressed on teaching concerns (t = −2.09, *p* = 0.04); however, teacher race/ethnicity was not (t = 1.55, *p* = 0.12). In Model 2, the overall regression equation was significant and accounted for 23% of the total variance of teaching concerns (F = 9.80, *p* < 0.001). When regressed on teaching concerns, dieting behavior (t = 0.42, *p* = 0.68), other weight control behaviors (t = −0.81, *p* = 0.42), self-efficacy (t = −1.66, *p* = 0.10), and number of years teaching (t = 0.50, *p* = 0.62) were all nonsignificant. However, teacher body esteem was significant (t = −6.59, *p* < 0.01) such that greater body esteem was related to significantly lower teaching concerns. Post hoc power analyses for multiple regression with seven predictors, an observed R^2^ of 0.228, a probability level of 0.05, and a sample of 259, yielded an observed statistical power of 0.999. 

### 3.4. Association of Teacher Variables and Program Implementation with Intervention Teachers

To examine the extent to which teacher characteristics and perceptions, and teaching concerns were associated with program implementation, another hierarchical regression was computed. In Model 1, teacher gender and ethnicity were regressed on the number of weeks teachers spent covering the program, accounting for 1% of the total variance of program implementation (F = 0.36; *p* = 0.70). Neither gender (t = −0.81; *p* = 0.42) nor race/ethnicity (t = −0.24; *p* = 0.81) was significantly associated with program implementation. In Model 2, the overall regression equation was nonsignificant and accounted for 10% of the total variance of the number of weeks spent covering the program (F = 1.33, *p* = 0.24). When regressed on program implementation, dieting behavior (t = 0.35, *p* = 0.73), other weight control behaviors (t = −0.85, *p* = 0.40), teaching concerns (t = 0.17; *p* = 0.87), number of years teaching (t = 1.36, *p* = 0.18), and body esteem (t = −1.04, *p* = 0.30) were all nonsignificant. However, teacher self-efficacy was significant (t = 2.56, *p* < 0.01) such that greater self-efficacy was related to significantly more program implementation. Post hoc power analyses for multiple regression with eight predictors, an observed R^2^ of 0.103, a 0.05 probability level, and a sample size of 124, yielded an observed statistical power of 0.743. 

## 4. Discussion

The current study aimed to address a gap in the literature by examining if teacher characteristics or perceptions were related to concerns teaching a positive body image program or the extent of program implementation. The goal was to illuminate potential barriers of implementation of universal school-based body image programs to facilitate program uptake and scalability. Across the 259 teachers in the study, there were some concerns reported with teaching a body image program to 4th and 5th graders; however, overall concern was relatively low. This may be due to sample characteristics. Teachers who enrolled in this study were those willing to participate in a randomized controlled trial where they would implement a body image program. This potentially creates a selection bias of teachers less hesitant to implement the program. An online survey of teachers, regardless of program implementation, may provide a greater diversity and intensity of concerns and may be a good avenue for future research. Regardless, over half of the teachers in the study reported some concerns with saying the wrong things, the extent to which their own body dissatisfaction may make them uncomfortable teaching the program, and inexperience teaching a body image program. Teachers’ written feedback reinforced concerns related to how their own body image could impact the program and also highlighted concerns with parents’ negative reactions to the program. 

Teacher body esteem was significantly associated with teacher concerns, such that higher body esteem scores were associated with fewer teacher concerns. This aligns with the teacher concerns expressed regarding teaching a body image program. Collectively, these findings suggest teacher manuals (i.e., facilitator guides) should incorporate a section on the impact of teacher body dissatisfaction on teaching a body image program. Providing information on important considerations and potential areas in the curriculum where a teacher’s body dissatisfaction may interfere may assuage concerns. For example, when assisting students in creating positive body affirmations, teachers can prepare example affirmations ahead of time. Similarly, when preparing for the lesson on combating body talk, diverse strategies may be outlined ahead of time. The teacher manual may also include sample statements and strategies to assist teachers. Further, future research should examine if teacher body esteem impacts the effectiveness of teaching a body image program. This may potentially help pacify potential anxiety and concerns or identify specific areas to include within teacher manuals. 

Although the Amazing Me curriculum is designed to not require any formal teacher training, online videos can be provided to teachers that address common concerns and pitfalls when teaching a body image program. This video can discuss common concerns and strategies and solutions for alleviating concerns. For other school-based body image programs that include teacher training, incorporating a section on anxiety and concerns and how to handle these, can be beneficial. Allowing teachers the space to voice their anxieties and concerns, and brainstorm ways to resolve these concerns may increase self-efficacy, uptake, and the number of activities implemented within the program. 

Teacher self-efficacy significantly predicted the number of weeks the teacher implemented the body image program. Higher self-efficacy was related with greater implementation. This is consistent with the existing literature. Research from school-based programs on psychological issues such as bullying and teasing, physical activity, and violence have all found teacher self-efficacy to predict program implementation [15,16,17,18]. It may be beneficial for school-based psychological programs to include a motivational interviewing approach within the training of teachers. Motivational interviewing is an evidence-based approach that explores and resolves ambivalence or anxiety/concerns, and focuses on motivational processes that facilitate behavior change [24]. This approach can increase teacher self-efficacy, increase motivation, highlight the importance and need of the program, and potentially increase program implementation. Indeed, the peer-reviewed journal *Prevention Science*, has a special issue dedicated to “Optimizing the Implementation and Effectiveness of Preventive Interventions through Motivational Interviewing”, which includes a discussion of core aspects of motivational interviewing, and how motivational interviewing incorporates into existing dissemination and implementation frameworks [25]. Further, the special issue includes empirical work on the Beliefs and Attitudes for Successful Implementation in Schools for Teachers (BASIS-T) program, which is a motivational interviewing session given to teachers in a group format immediately before and after teacher training on an evidence-based prevention program on teachers [26]. The use of this program increased teacher motivation and self-efficacy to increase program implementation.

It is important to note that gender was associated with teaching concerns but not program implementation. Male teachers reported more concerns with teaching a body image program than female teachers. Specifically, male teachers expressed significantly more worry about saying the wrong things than female teachers. Unfortunately, it is unclear if teaching concerns qualitatively differed across the genders as no male teachers provided written feedback. In addition, there were only nine male teachers in the sample, limiting the generalizability of the findings. Future research should consider including a larger sample size of diverse gender identities to explore teaching concerns and implementation of a body image program. 

Another noteworthy consideration is the fact that the Amazing Me program includes appearance- and weight-related bullying and teasing, yet teachers reported no concerns related to teaching bullying and teasing prevention. This may be because all 50 states address bullying and teasing in schools, with some states requiring anti-bullying prevention programs [27]. Thus, teachers may have experience and confidence in implementing anti-bullying activities. 

Aside from the strengths and limitations of the current study mentioned above, the current study has a couple more notable strengths and limitations. The sample was large and geographically diverse representing over 30 states across the United States and schools across various socioeconomic statuses. However, data collection occurred during the COVID-19 pandemic, which presented unprecedented stressors for teachers, which could have influenced the recruitment, retention, and results of the study. The online survey administered to teachers at baseline was face valid, and teachers could easily determine why the authors would be asking about teacher dieting, weight control behaviors, body esteem, and teaching self-efficacy, prior to the potential implementation of the body image program. It is unclear to the extent that demand characteristics could have played a role in teacher responses. Finally, the implementation variable consisted of the number of weeks a teacher implemented the program, potentially limiting understanding of the ‘dose effect’ of program implementation. It is possible that teachers implemented one, two, or three activities per week and this is not captured in the data. 

## 5. Conclusions

The current study addressed a gap in the literature by examining the extent to which teacher characteristics and perceptions relate to teaching concerns and implementation of a school-based body image program. Currently, there are numerous school-based body image programs that have been developed, but a paucity of empirical research examining factors influencing uptake and implementation. Teacher gender and body esteem were related to teaching concerns, suggesting that male gender and body dissatisfaction are important to address in training and teacher manuals for the program. Consistent with existing research for other psychological issues, teacher self-efficacy was related to the number of weeks a teacher implemented the program. Spending time increasing teacher self-efficacy and motivation during training may potentially yield greater uptake and implementation efforts by teachers. Given the paucity of existing research, the findings from this study require replication. School-based body image- and appearance-related anti-bullying and teasing programs have the potential to increase psychological well-being in youth, in a scalable and cost-effective manner. Thus, exploring factors that influence implementation can be a worthy pursuit for the body image field.

## Figures and Tables

**Table 1:** Sociodemographic Characteristics of Teachers at Baseline.

Teacher Characteristics	*n*	%
Age ^1^		
22–29	22	8.6
30–39	83	32.1
40–49	85	32.7
50–59	50	19.8
60–70	12	4.8
Gender Identity		
Male	9	3.5
Female	232	89.6
Gender Non-Conforming	1	0.4
Prefer not to Answer/Not Listed	17	6.6
Race/Ethnicity		
Asian or Asian American	8	3.1
Black or African American	30	11.6
Hispanic or Latino/a/e	16	6.2
Multiracial	11	4.2
Other/Not Specified	13	5.0
White or Caucasian	179	69.1
Number of Years Teaching ^1^		
0–9	107	41.3
10–19	91	35.1
20–29	45	1.2
30–37	6	2.4

^1^ The number and % of missing data for age is 7 (2.7%) and 10 (3.9%) for number of years teaching. No other variables in the Table had missing data.

**Table 2:** Correlations among Study Variables at Baseline.

Variable	1	2	3	4	5	6	7	8	9
1. General Self-Efficacy									
2. Weight Control Behaviors	0.03								
3. Dieting Behaviors	0.02	0.51 ^**^							
4. Body Esteem Scale Adult and Adolescents	0.04	−0.52 ^**^	−0.35 ^**^						
5. Number of years teaching	0.11	0.06	0.18 ^**^	0.02					
6. Teaching Concerns	−0.16 ^**^	0.20 ^**^	0.16 ^**^	−0.43 ^**^	0.02				
7. Gender	−0.05	−0.05	−0.04	0.02	0.04	0.06			
8. Ethnicity	−0.09	0.01	−0.05	0.04	−0.04	0.11	−0.02		
9. # of Weeks Program Implemented	0.25 ^**^	−0.02	−0.02	−0.10	−0.13	0.01	−0.08	−0.02	
*M (SD)*	4.28 (0.61)	3.00 (2.65)	2.60 (0.73)	3.21 (0.67)	12.23 (7.93)	1.52 (0.44)	2.12 (0.97)	0.09 (0.29)	4.90 (1.45)

** *p* < 0.01.

**Table 3:** Frequency of Concerns Endorsed for each Item on the Teachers Concerns Measure.

Teacher Concerns Items N (%)	Not At All Concerned	A Little Concerned	Moderately Concerned	Very Concerned
I worry I may say the wrong thing.	91 (35.1)	133 (51.4)	34 (13.1)	1 (0.4)
I feel I do not know enough about body image to teach this curriculum.	144 (55.6)	92 (35.5)	21 (8.1)	2 (0.8)
I’ve never done anything like this before and I don’t know what it will be like.	118 (45.5)	106 (40.9)	32 (12.4)	3 (1.2)
I worry that others will find out that I am dissatisfied with my body.	218 (84.1)	30 (11.6)	8 (3.1)	3 (1.2)
I sometimes feel dissatisfied about parts of my body and this makes me feel uncomfortable.	114 (44.0)	104 (40.2)	36 (13.9)	5 (1.9)
Talking about body image makes me feel uncomfortable.	197 (76.0)	53 (20.5)	7 (2.7)	2 (0.8)
Discussing body image with 4th and 5th graders seems difficult.	164 (63.3)	79 (30.5)	15 (5.8)	1 (0.4)

Note: N = 259.

**Table 4:** Regressions with Teaching Concerns and Program Implementation.

		95% CI					
Measure	B	*LL*	*UL*	SE B	β	R^2^	Adj. R^2^
1. Teaching Concerns							
Model 1 *						0.030	0.030
Gender	−0.12	−0.23	0.01	0.06	−0.13		
Race/Ethnicity	0.15	−0.04	0.34	0.10	0.10		
Model 2 **						0.228	0.205
Dieting Behaviors	0.02	−0.06	0.10	0.04	0.03		
Weight Control Behaviors	−0.01	−0.03	0.01	0.01	−0.06		
Body Esteem **	−0.30	−0.39	−0.21	0.05	−0.45		
Self-Efficacy	−0.26	−0.56	0.05	0.15	−0.10		
Number of Years Teaching	0.00	−0.01	0.01	0.00	0.03		
2. # Weeks Implementing Program							
Model 1						0.007	−0.013
Gender	−0.09	−0.29	0.12	0.11	−0.08		
Race/Ethnicity	−0.03	−0.27	0.21	0.12	−0.02		
Model 2						0.103	0.026
Dieting Behaviors	0.10	−0.45	0.64	0.28	0.05		
Weight Control Behaviors	−0.06	−0.21	0.08	0.07	−0.12		
Body Esteem	−0.30	−0.88	0.28	0.29	−0.13		
Self-Efficacy **	0.63	0.14	1.12	0.25	0.26		
Number of Years Teaching	−0.03	−0.06	0.01	0.02	−0.14		
Teaching Concerns	0.06	−0.68	0.81	0.38	0.02		

Note. Gender is dummy coded with 0 representing males and 1 representing females. CI = confidence interval; UL = upper limit; LL = lower limit. N = 259 for hierarchical regression with teacher concerns. N = 124 intervention teachers that reported the number of weeks implementing the program in the second hierarchical regression. * *p* < 0.05. ** *p* < 0.01.

## Data Availability

The data presented in this study are available on request from the corresponding author and subject to IRB approval. The data are not publicly available due to privacy and ethical restrictions.
